# The BK_Ca_ (*slo*) channel regulates the cardiac function of Drosophila

**DOI:** 10.14814/phy2.15996

**Published:** 2024-04-01

**Authors:** Shubha Gururaja Rao, Alexander Lam, Sarah Seeley, Jeniffer Park, Shriya Aruva, Harpreet Singh

**Affiliations:** ^1^ Department of Pharmaceutical and Biomedical Sciences The Raabe College of Pharmacy, Ohio Northern University Ada Ohio USA; ^2^ Department of Physiology and Cell Biology The Ohio State University Columbus Ohio USA

**Keywords:** antioxidants, BK channels, life span, mitochondria, potassium channel, reactive oxygen species

## Abstract

The large conductance, calcium, and voltage‐active potassium channels (BK_Ca_) were originally discovered in *Drosophila melanogaster* as *slowpoke* (*slo*). They are extensively characterized in fly models as ion channels for their roles in neurological and muscular function, as well as aging. BK_Ca_ is known to modulate cardiac rhythm and is localized to the mitochondria. Activation of mitochondrial BK_Ca_ causes cardioprotection from ischemia–reperfusion injury, possibly via modulating mitochondrial function in adult animal models. However, the role of BK_Ca_ in cardiac function is not well‐characterized, partially due to its localization to the plasma membrane as well as intracellular membranes and the wide array of cells present in mammalian hearts. Here we demonstrate for the first time a direct role for BK_Ca_ in cardiac function and cardioprotection from IR injury using the *Drosophila* model system. We have also discovered that the BK_Ca_ channel plays a role in the functioning of aging hearts. Our study establishes the presence of BK_Ca_ in the fly heart and ascertains its role in aging heart function.

## 1 INTRODUCTION

The calcium (Ca^2+^) and voltage‐activated large conductance potassium channel (MaxiK/*slo*/BK_Ca_) was originally identified in *Drosophila* at the *slowpoke* (*slo*) locus (Atkinson et al., 1991; Singh & Wu, 1989). In mammals, BK_Ca_ is encoded by the *Kcnma1* gene (Singh, Stefani, & Toro, 2012). The BK_Ca_ channel is expressed in the plasma membrane of all eukaryotic cells, where it forms a tetrameric potassium channel consisting of a large pore‐forming α‐subunit with >1250 amino acids (Toro et al., 2014). BK_Ca_ channels are implicated in regulating circadian rhythms, cellular excitability, bladder function, smooth muscle contraction, and cardiac physiology (Ancaten‐Gonzalez et al., 2023). Abnormal expression and mutations in the *Kcnma1* gene are associated with several pathological conditions and are termed *Kcnma1*‐linked channelopathies (Meredith, 2023). These include autism, epilepsy, dyskinesis, intellectual disability, neurodevelopmental disorder, ataxia, cerebral and cerebellar atrophy, and bone thickness (Miller et al., 2021).

In the heart, the expression of BK_Ca_ is vital for cardiac rhythm by regulating the function of SA node cells (Imlach et al., 2010; Lai et al., 2014). In mammalian adult cardiomyocytes, BK_Ca_ channels are exclusively present in the inner mitochondrial membrane (Singh et al., 2013; Szabo & Szewczyk, 2023). In the inner membrane of mitochondria, activation of the BK_Ca_ channel results in cardioprotection from ischemia–reperfusion (IR) injury (Singh et al., 2013; Szteyn & Singh, 2020). Surprisingly, in neonates, these channels are not exclusively present in the mitochondria of cardiomyocytes (Sanghvi et al., 2022). They are also present in the plasma membrane, and their activation results in adverse myocardial infarction outcomes (Sanghvi et al., 2022). In *Drosophila*, where BK_Ca_ shares 60% homology to human BK_Ca_, inactivation of BK_Ca_ was shown to slow the heartbeat and produce fibrillatory cardiac contractions (Pineda et al., 2021). In *Drosophila* larvae, there was a large variability in the heart rate in the saline solution; application of 2‐Aminoethyl diphenylborinate (2‐APB) decreased the heart rate in the *slo* mutant but increased the heart rate in wild‐type strains (Hensley et al., 2023). In an independent study (Pineda et al., 2021), adult‐only *slo* knockdown using HandGS‐Gal4 was sufficient to cause cardiac abnormalities. There is a clear indication of the role of BK_Ca_ in heart function during development, but the precise role is not yet established.

In *Drosophila*, BK_Ca_ was shown to carry transient Ca^2+^‐dependent K^+^ currents in neurons (Saito & Wu, 1991) and muscle cells (Komatsu et al., 1990; Singh & Wu, 1989). Recently, *slo* was also implicated in circadian rest: activity, rhythm, strength, and exerting effects in multiple components of the circadian circuit (Ruiz et al., 2021). Our results also implicate *slo* in the shorter lifespan phenotype in flies, which was partially attributed to abnormal reactive oxygen species (ROS) production and mitochondrial function (Gururaja Rao et al., 2019). We have also shown that in mice and *Drosophila*, BK_Ca_ is present in the inner membrane of mitochondria (Gururaja Rao et al., 2019; Singh et al., 2013). The absence of the protein in *slo* mutants disrupted the cristae of mitochondria and resulted in the swelling of mitochondria (Gururaja Rao et al., 2019). The accelerated aging phenotype makes *slo* mutant an appropriate model to establish the role of BK_Ca_ in an aging heart.

In the work presented here, we studied the role of BK_Ca_/*slo* in cardiac function in an age‐dependent manner. We established that ablation of BK_Ca_ reduced heart rate and cardiac function as flies aged. We also deciphered that hypoxia‐reoxygenation has a detrimental impact on hearts lacking *slo*. Our results also show abnormalities in the cardiac fiber size, supporting the observations of cardiac function abnormalities. Taken together, our results define a direct role of the BK_Ca_ channel in aging hearts and hypoxia‐reoxygenation.

## 2 MATERIALS AND METHODS

### 2.1 *Drosophila* stocks, reagents, dyes, and antibodies

All *Drosophila melanogaster* stocks were maintained at 25°C on standard medium (jazz mix, without Nipagin) unless otherwise stated. The Canton S strain served as the wild‐type (wt) stock and is indicated as ‘wt’ throughout the manuscript. The *slo^1^* mutants and Canton S were obtained from the Bloomington Stock Centre (Indiana University, USA).

### 2.2 Immuno‐cytochemistry

The cardiac tube was dissected and fixed with 4% (*w/v*) paraformaldehyde [PFA (Sigma, P6148)], washed, and permeabilized with 0.4% (*v/v*) triton‐X100. Tissues were blocked with normal goat serum (10%, *v/v*) and stained with primary antibodies [anti‐BK_Ca_ 1:100, Neuro mAb, clone L6/60] and secondary antibodies (cell signaling – 8890, 8890, 4412, 4408), followed by DAPI (Sigma‐Aldrich, D9542) for tissues.

### 2.3 Dihydroethidium (DHE) staining

*Drosophila* larval hearts were dissected quickly and placed in DHE (Molecular Probes, D11347) in PBS (1:1000 dilution) for 5 min, and then washed in PBS three times for 3 min each. The hearts were slightly fixed in 4% (*w/v*) PFA for 3 min and then washed again in PBS twice for 2 min each time. Hearts were then mounted in PBS and immediately photographed on an Olympus confocal microscope using the FLUOVIEW FV1000 [20×, 40×, and 60× (1.4 NA) oil objectives] (*n* = 3 independent experiments).

### 2.4 Cardiac function measurements

The flies were selected at designated ages and were exposed to continuous CO_2_ for 30 s to 1 min; the flies were placed on a thin layer of grease and placed with their dorsal side facing toward the Optical Coherence Tomography (OCT) microscope (Thorlabs) (Lam et al., 2018). The flies were placed under the OCT system for image acquisition.

The TELESTO‐II OCT system (Thorlabs, Munich, Germany) with a nominal wavelength of 1310 nm was used to obtain 2D B‐mode and 2D M‐mode images through the ThorImage OCT 4.4.6.0 software. The procedure described earlier (Lam et al., 2018; Wolf et al., 2011) was used to locate the cardiac tube using 2D B‐mode images, then switching to 2D M‐mode images to continuously record the cardiac cycle. The B‐mode 2D images had an axial scan (A‐scan) line rate of 76 kHz, and the M‐mode 2D images had an A‐scan line rate of 5.5 kHz.

The 2D M‐mode images were used to quantify the cardiac function. The end‐diastolic diameter (EDD) and end‐systolic diameter (ESD) were measured and recorded by determining the distances between the superior and inferior walls of the cardiac tube in mid‐diastolic and mid‐systolic periods throughout the normal cardiac cycles of each fly. The averages were taken for the EDD and ESD dimensions of each fly; the average EDD and ESD were used to calculate the fractional shortening defined as ((EDD − ESD)/ESD) × 100. The heart rate was determined from the M‐mode images obtained.

### 2.5 Measurement of fiber thickness

Actin was labeled with a phalloidin reagent, as described earlier (Karekar et al., 2021). In brief, cardiac tubes were carefully dissected from third‐instar larvae, washed with PBS, and fixed in ice‐cold 4% (*w/v*) paraformaldehyde (PFA). Cardiac tubes were washed and permeabilized with 0.4% (*v/v*) Triton‐X100. Cardiac tissues were blocked with 10% (*v/v*) normal goat serums and stained overnight at 4°C using Phalloidin‐iFluor 488 Reagent (Abcam, ab176753). Images of each sample were captured at 0.5 μm with an Olympus confocal microscope using the FLUOVIEW FV1000 [20×, 40×, and 60× (1.4 NA) oil objectives]. Fiji Image J software (NIH, Bethesda, MD, USA) was used to find the diameters of 40–60 fibers per tissue sample (*n* = 3 for wild type and n = 3 *slo* mutants), and data were combined from all samples of the same group and plotted as a frequency histogram with a bin size of 0.05 μm. Average sizes were also plotted, and student two‐tailed *t*‐tests were performed to test the significance.

### 2.6 Hypoxia‐reperfusion injury in *Drosophila*

Hypoxia reperfusion injury in *Drosophila* was achieved by incubating flies in a hypoxia chamber (stem cell technologies) for 30 min at 1 percent oxygen (nitrogen replacement), followed by 60 min of reoxygenation in room air. Wild‐type and *slo* mutants were collected at 30 min for experiments (hypoxia time point) or continued with reoxygenation and collected at the endpoint. Flies were either dissected for immunofluorescence studies or heart function was measured as described above.

## 3 RESULTS

### 3.1 Role of BK_Ca_ in cardiac function

**FIGURE 1:**
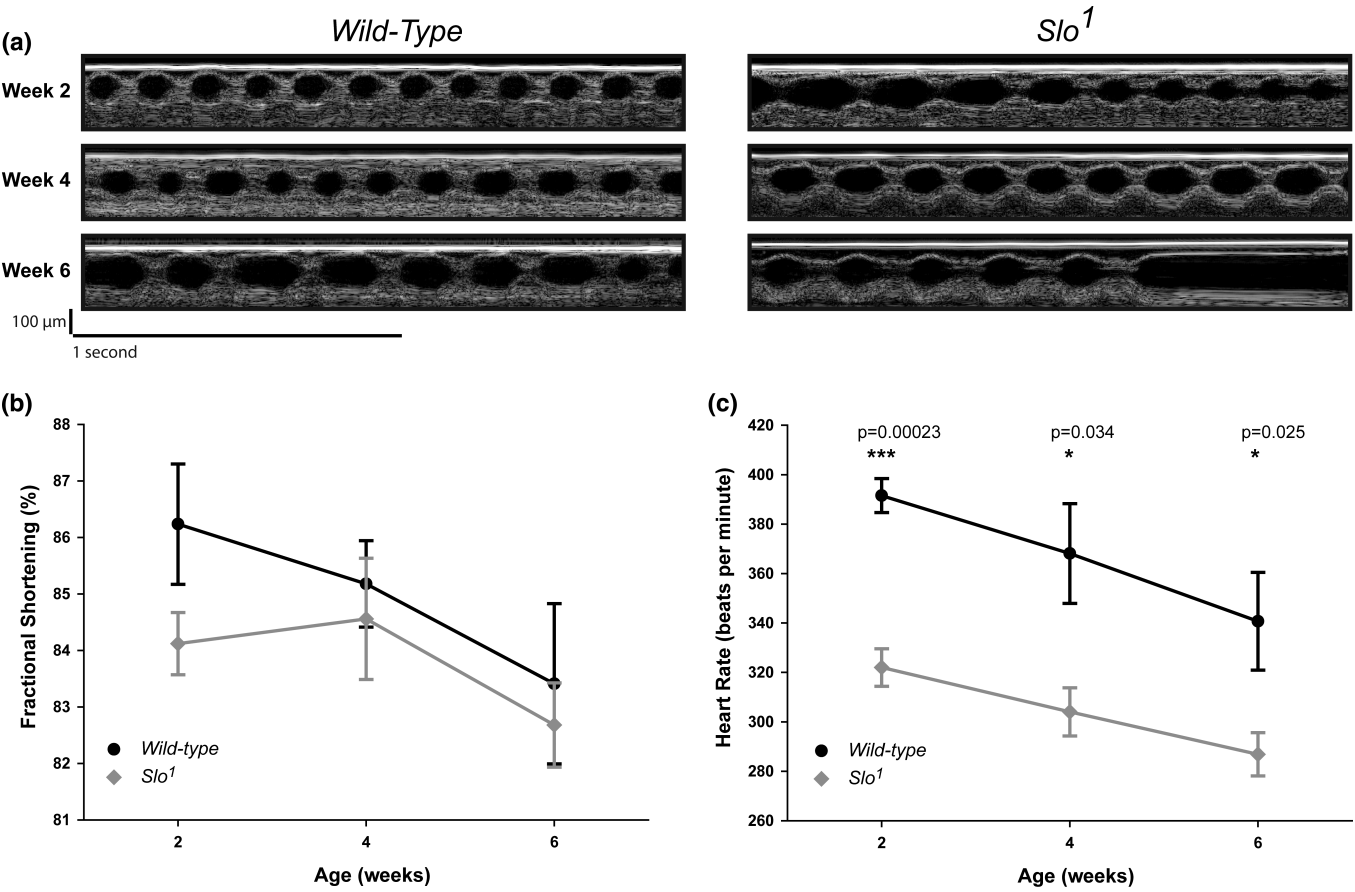
Cardiac function of wild‐type and *slo* mutant flies. (a) Cardiac function of *Drosophila* was evaluated by optical coherence tomography (OCT). Continuous images were obtained for wild‐type and *slo* mutant flies at week 2, week 4, and week 6. Note irregular beating in week 6 in *slo* mutant flies. (b) Fractional shortening analysis of wild‐type and *slo* mutant flies at week 2, week 4, and week 6. (c) Analysis of heart rate obtained from OCT images for wild‐type and *slo* mutant flies at week 2, week 4, and week 6. (*n* = 25–30 flies in each group). *p* Values for the data reported were calculated using a two‐tailed *t*‐test.

We have previously shown that the presence of BK_Ca_ determines the life span of *Drosophila* (Gururaja Rao et al., 2019). However, the direct role of BK_Ca_ in cardiac function is not yet completely established. We took advantage of the accelerated aging phenotype of the *slo* mutant (Gururaja Rao et al., 2019) and evaluated cardiac function using OCT in fully intact, non‐anesthetized flies (Lam et al., 2018). We evaluated the cardiac function of *slo* mutants (25 adult flies) and corresponding wild‐type (30 flies) flies at weeks 2, 4, and 6 after eclosion. End‐diastolic and end‐systolic measurements were obtained from the videos obtained by OCT. Based on these measurements, cardiac fractional shortening (FS), a direct measure of the contractile strength of the cardiac tube, was obtained. As shown in figure 1, at week 2, fractional shortening (FS) of *slo* mutants was slightly (but significantly) lower than the age‐matched wild‐type flies. However, with age, there was a significant drop in FS for wild‐type flies but not in *slo* mutants. Therefore, at weeks 4 and 6, we did not detect any difference in FS between the two groups (Figure 1).

BK_Ca_ is known to play a role in the regulation of heart rate (Singh, 2021). Genetic (Imlach et al., 2010; Lai et al., 2014) or pharmacological inhibition (Patel et al., 2018) indicates that the absence of BK_Ca_ causes a reduction in the heart rate. Here, we evaluated whether BK_Ca_ plays a role in the regulation of heart rate during aging in *Drosophila*. In agreement with mammalian data, the *slo* mutant showed a 20% reduction in heart rate at week 2 as compared to wild‐type flies. Unlike FS, the difference between HR also remains significantly lower at weeks 4 and 6. There was a significant drop (15%) in the heart rate of wild‐type male flies from weeks 2 to 6. The drop for *slo* mutant male flies was 8%. There was no difference between the cardiac function of *slo‐* or wild‐type males and female flies in FS during aging. In *slo* mutant, we did observe arrhythmias from week 2 onwards to week 6. Taken together, our results indicate that *slo* plays an active role in maintaining the cardiac function of an aging fly heart.

### 3.2 Localization of BK_Ca_ in fly heart

**FIGURE 2:**
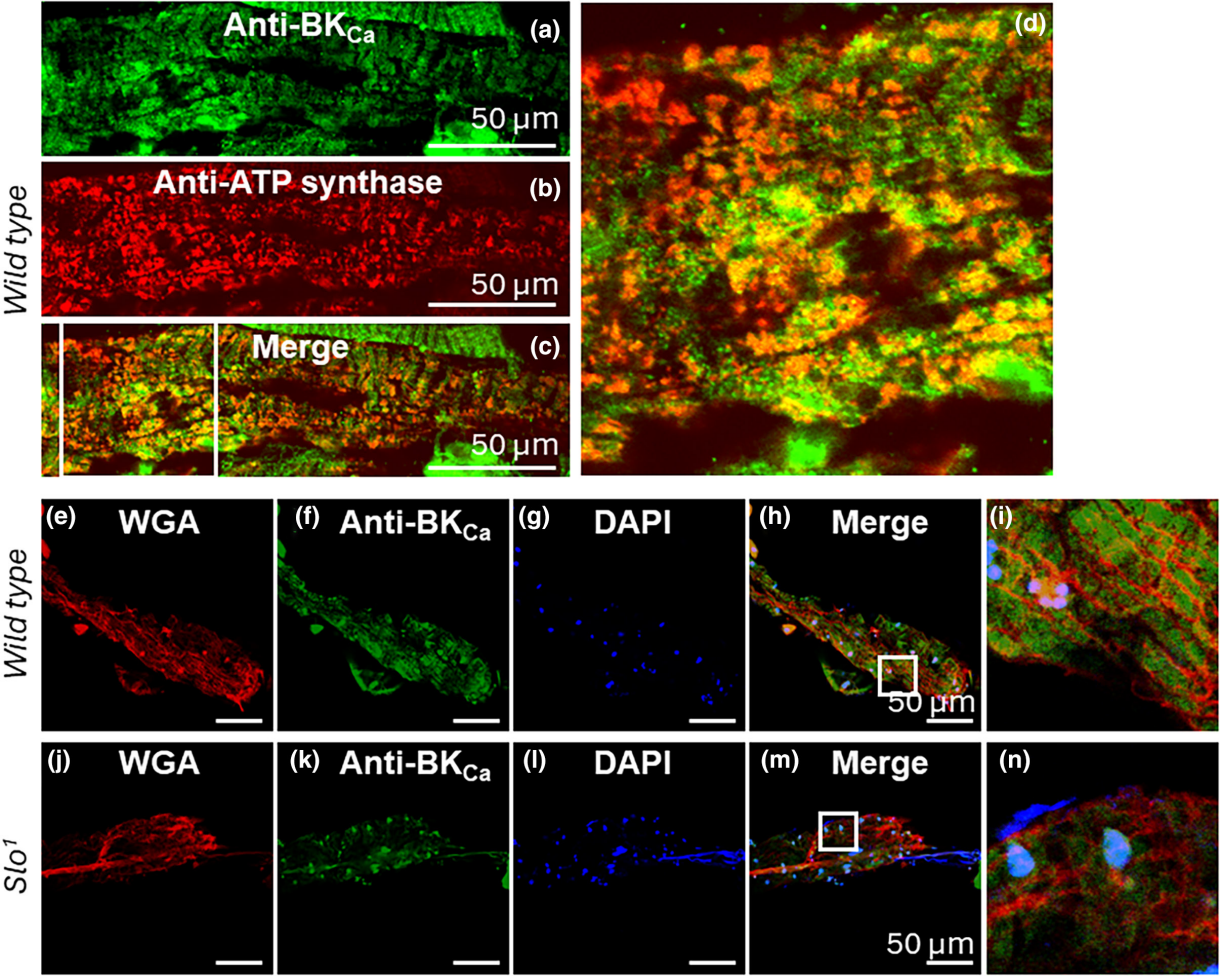
Localization of BK_Ca_ in the *Drosophila* heart. (a–d) Cardiac tubes were isolated and labeled with anti‐BK_Ca_ (a) and anti‐ATP synthase (for mitochondria, b). (c) Merge images of (a) and (b, d) enlarged image from a boxed region in (c). (e–i). Cardiac tubes labeled with anti‐BK_Ca_ and wheat germ agglutinin (WGA, plasma membrane marker) isolated from wild‐type flies. (e) Cardiac tube labeled with WGA. (f) Cardiac tube labeled with anti‐BK_Ca_. (g) Cardiac tube stained for nucleus with DAPI. (h) Merged image of (e, f) and (g, i) Enlarged region from a white box in (h) BK_Ca_ does not localize to the WGA labeled plasma membrane. (j–n) Cardiac tubes labeled with anti‐BK_Ca_ and WGA isolated from *slo* mutant flies. (j) Cardiac tube labeled with WGA. (k) Cardiac tube labeled with anti‐BK_Ca_ showed no significant labeling for BK_Ca_. (l) Cardiac tube stained for nucleus with DAPI. (m) Merged image of (j, k) and (l, n) Enlarged region from a white box in m.

Since BK_Ca_ plays a role in the cardiac function of *Drosophila*, we evaluated its localization in the heart tube. Cardiac tubes were isolated and labeled with anti‐BK_Ca_ antibodies described earlier (Gururaja Rao et al., 2019; Singh et al., 2013) and anti‐ATP synthase antibodies. As shown in Figure 2, BK_Ca_ is present in the fly heart, where it localizes with the mitochondria (52.7 ± 5%, *n* = 10), as calculated using PPI, protein proximity index (Singh, Lu, et al., 2012; Zinchuk et al., 2011). This is in agreement with other animal models where BK_Ca_ is present in the cardiac mitochondria and our recently published data where BK_Ca_ was shown to be present in mitochondria and plays a role in its function (Goswami et al., 2018; Singh et al., 2013; Xu et al., 2002). We also tested whether the plasma membrane of cardiac cells also possesses BK_Ca_. We labeled the plasma membrane of cardiac cells from wild‐type and *slo* mutants with wheat germ agglutinin (WGA, Figure 2e,j). Cardiac tubes were incubated with anti‐BK_Ca_ antibodies (Figure 2f,k), and nuclei were labeled with DAPI (Figure 2g,l). The labeling for BK_Ca_ in *slo* mutants was not observed (Figure 2f,k). However, the specific signal for BK_Ca_ in the wild‐type cardiac type was found to be present inside the cardiac cells labeled with WGA (Figure 2h,i). Similar to mammalian hearts, in *Drosophila* cardiomyocytes, BK_Ca_ localizes mainly to the mitochondria.

### 3.3 The absence of BK_Ca_ results in increased ROS and thickening of Actin fibers

**FIGURE 3:**
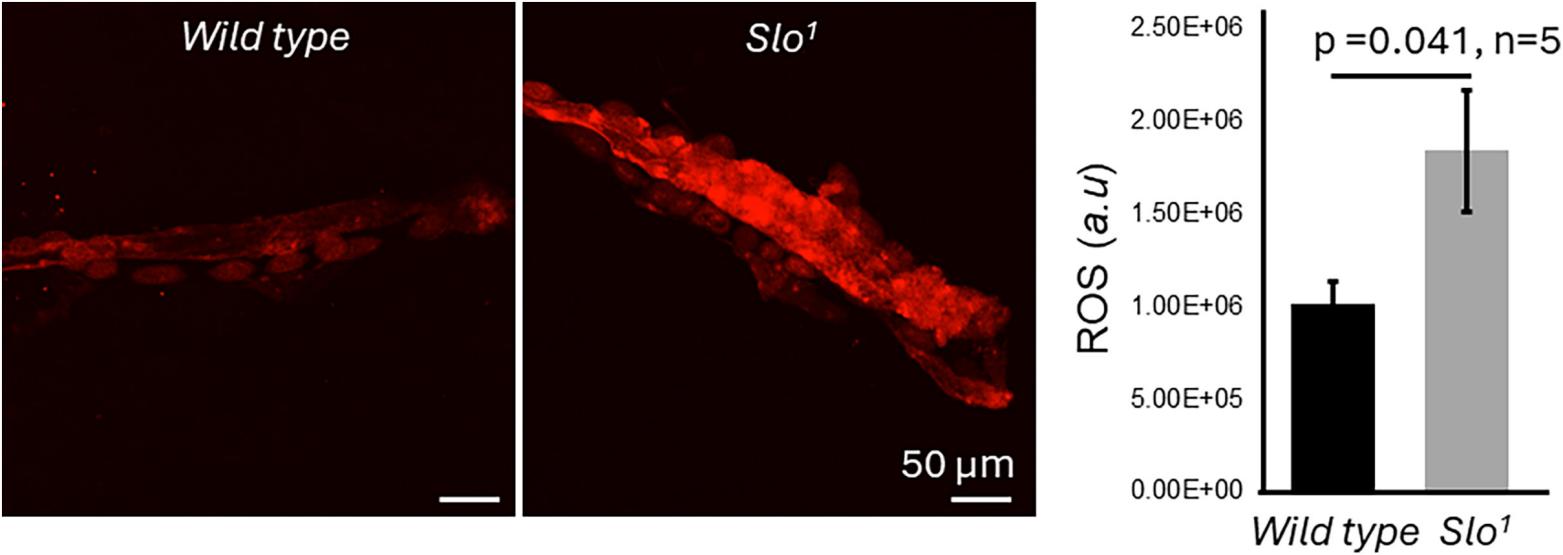
ROS production in the *Drosophila* heart. ROS production was estimated by staining freshly isolated cardiac tubes from wild‐type and slo mutant flies with the DHE dye and imaging them at the same settings. The *slo* mutant showed a higher amount of staining of ROS as compared to the wild‐type flies (*n* = 3); the fluorescence intensity was quantified using Image J (FIJI) software. Bar graphs represent the quantification of ROS production from the fly heart.

**FIGURE 4:**
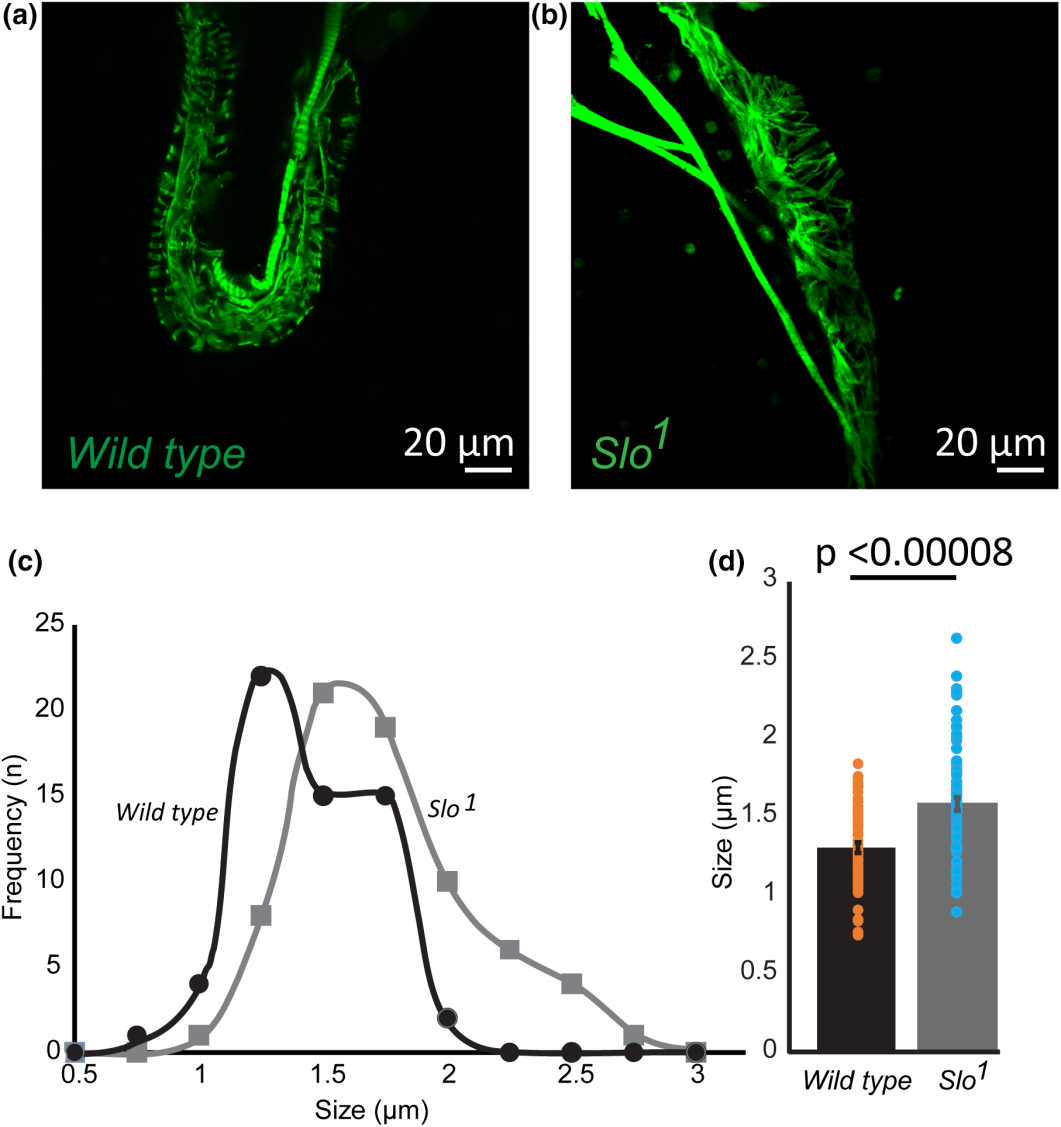
*Slo* mutants have abnormal cardiac fibers. Cardiac tubes from larvae were isolated and stained with phalloidin to label Actin filaments in wild‐type (a) and *slo* mutant (b). Stained Actin filaments were measured using Image J. (c) Fiber thickness was obtained, and a frequency histogram was plotted for wild‐type and *slo* mutant flies. The *slo* mutant flies showed increased fiber thickness. (d) Bar graphs indicate that *slo* mutant flies had thick Actin filaments as compared to the wild‐type flies (*n* = 5 flies).

Similar to what we observed in our previous studies in muscles (Gururaja Rao et al., 2019), where we found an increase in ROS in the absence of BK_Ca_, we also found that ROS increased significantly in the *Drosophila* hearts, as shown by DHE staining, a dye that detects ROS (*p* < 0.05, *n* = 3; Figure 3). *Drosophila* is increasingly used for the hypertrophic cardiomyopathy model as it provides an integrative genetic tool to understand the mechanism of the disease. Since we observed abnormal cardiac function, heart rate, and elevated ROS production, we measured the cardiac fibers labeled with phalloidin. The cardiac fibers in *slo* third instar larvae were significantly (*p* < 0.00000082, *n* = 45–60 fibers, *n* = 3–5 flies) thicker than wild‐type flies (Figure 4). The cardiac fibers in wild‐type flies were thick and thin in distribution, but the *slo* fibers were similar to the thick ones of wild‐type flies (Figure 4). These results provide a possible mechanism for cardiac dysfunction that involves oxidative stress and abnormal extracellular matrix meshwork; this phenomenon is found in hypertensive and cardiomyopathy conditions in human hearts (Hoshino et al., 1983; Karekar et al., 2021).

### 3.4 Role of BK_Ca_ in protecting the fly heart

We have discovered that BK_Ca_ plays a role in fly heart function (Figure 1), and earlier, our group and others have shown that BK_Ca_ is involved in protecting the mouse heart from hypoxia‐reperfusion injury (Frankenreiter et al., 2017, 2018; Goswami et al., 2018; Sanghvi et al., 2022; Sato et al., 2005; Singh et al., 2013; Stumpner et al., 2012; Szteyn & Singh, 2020). Here, we investigated whether *slo* plays a role in protecting the heart from ischemia–reperfusion injury. We also segregated them into males and females for this experiment. We evaluated the cardiac function of 2‐week‐old wild‐type and *slo* mutant flies before subjecting them to hypoxia and reperfusion. Each fly after the cardiac evaluation was transferred to a hypoxia chamber (1% O_2_) for 30 min. Cardiac function was evaluated immediately after removal from the hypoxia chamber, and another measurement was taken after 60 minutes of incubation in room air (21% O_2_).

**FIGURE 5:**
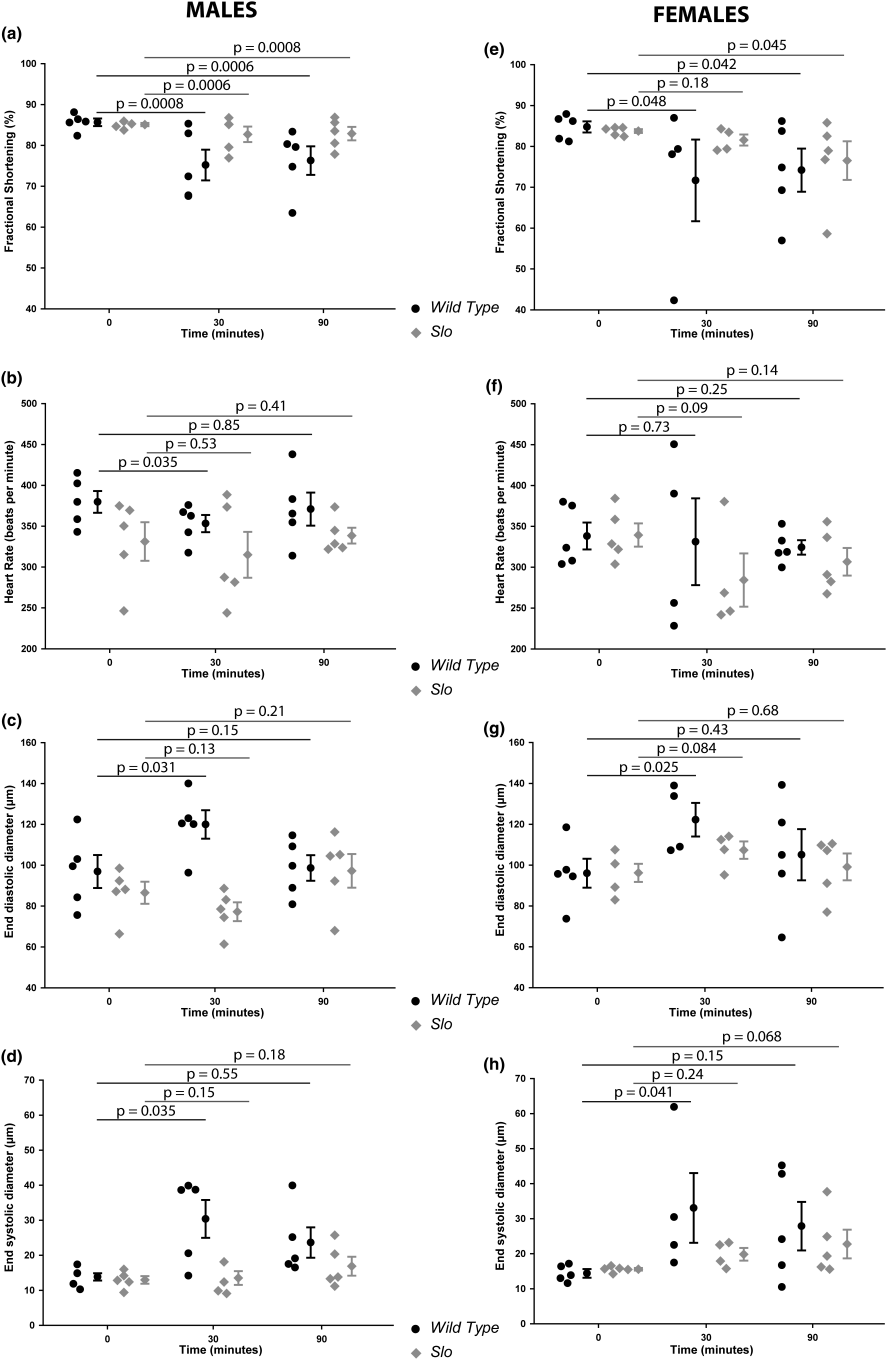
Cardiac functional evaluation after hypoxia and reoxygenation. Adult wild‐type and *slo* mutant flies were subjected to hypoxia and reoxygenation. (a–d) Cardiac function of male wild‐type and *slo* mutant flies. Fractional shortening (a), heart rate (b), end‐diastolic diameter (c), and end‐systolic diameter (d) were calculated for male wild‐type (black) and *slo* mutant (gray) flies. (e–h) Cardiac function of female wild‐type and *slo* mutant flies. Fractional shortening (e), heart rate (f), end‐diastolic diameter (g), and end‐systolic diameter (h) were calculated for male wild‐type (black) and *slo* mutant (gray) flies. *p* Values for the data reported were calculated using two‐tailed *t*‐tests.

As shown in Figure 5, wild‐type males and females showed a significant (*p* < 0.001, *n* = 6, and *p* < 0.05, *n* = 5, respectively) reduction in fractional shortening immediately after hypoxia (Figure 5a,e). After 60 minutes of reperfusion, fractional shortening failed to recover in both male and female flies. In *slo* mutant flies, males showed a significant reduction (*p* < 0.001, *n* = 5) in fractional shortening after hypoxia as compared to the fractional shortening before the onset of hypoxia. Similar to wild‐type flies, *slo* mutant males failed to recover after 60 min of reperfusion. Though *slo* male mutants showed a reduction in fractional shortening after 30 min of hypoxia as well as 90 min (30 min hypoxia and 60 min reoxygenation) of hypoxia and reoxygenation, the decrease in cardiac function was not as severe as in wild‐type flies. Interestingly, in female flies, a reduction in the protection of fractional shortening was seen immediately after hypoxia, but there was no difference in fractional shortening when compared to wild‐type flies after hypoxia and reoxygenation for 60 min.

BK_Ca_ is well‐characterized for its role in the regulation of adult rodent and murine heart rates (Imlach et al., 2010; Lai et al., 2014). We also evaluate whether BK_Ca_ plays a role in the regulation of heart rate in *Drosophila*. As shown in Figure 5b,f, there was a significant reduction (*p* < 0.05, *n* = 5) in heart rate in male mutants as compared to the wild type. At 30 min of hypoxia, heart rate decreased significantly in wild‐type male flies but not in *slo* mutants. After 90 min of hypoxia‐reoxygenation, the heart rate recovered to pre‐hypoxia levels in both wild‐type and *slo* mutant flies. Intriguingly, there was no difference noted in the heart rate of female *slo* mutant flies at pre‐hypoxia, after hypoxia, and after hypoxia‐reoxygenation. Female *slo* mutant flies only showed a slight decrease in heart rate as compared to the wild type after 30 min of hypoxia and 90 min of hypoxia‐reoxygenation.

We also evaluated the dimensions of the cardiac tubes of wild‐type and *slo* mutant flies. Even though *slo* mutant flies are smaller in size, the cardiac tube end‐diastolic diameter (Figure 5e, g) and end‐systolic diameter (Figure 5d, h) were similar in male and female wild‐type as well as *slo* mutant flies. After 30 min of hypoxia, the end‐diastolic diameter of both male and female wild‐type flies increased, but *slo* mutants (both sexes) presented no increase in dimensions. After 90 min of hypoxia‐reoxygenation, there was a difference found in both wild‐type and *slo* mutant male and female flies. Similar to end‐diastolic diameter, end‐systolic diameter significantly increased in wild‐type male and female flies but not in *slo* mutant flies. After 90 min of hypoxia and reoxygenation, there was no change in the diameter of *slo* mutants or wild‐type male or female flies.

## 4 DISCUSSION

**FIGURE 6:**
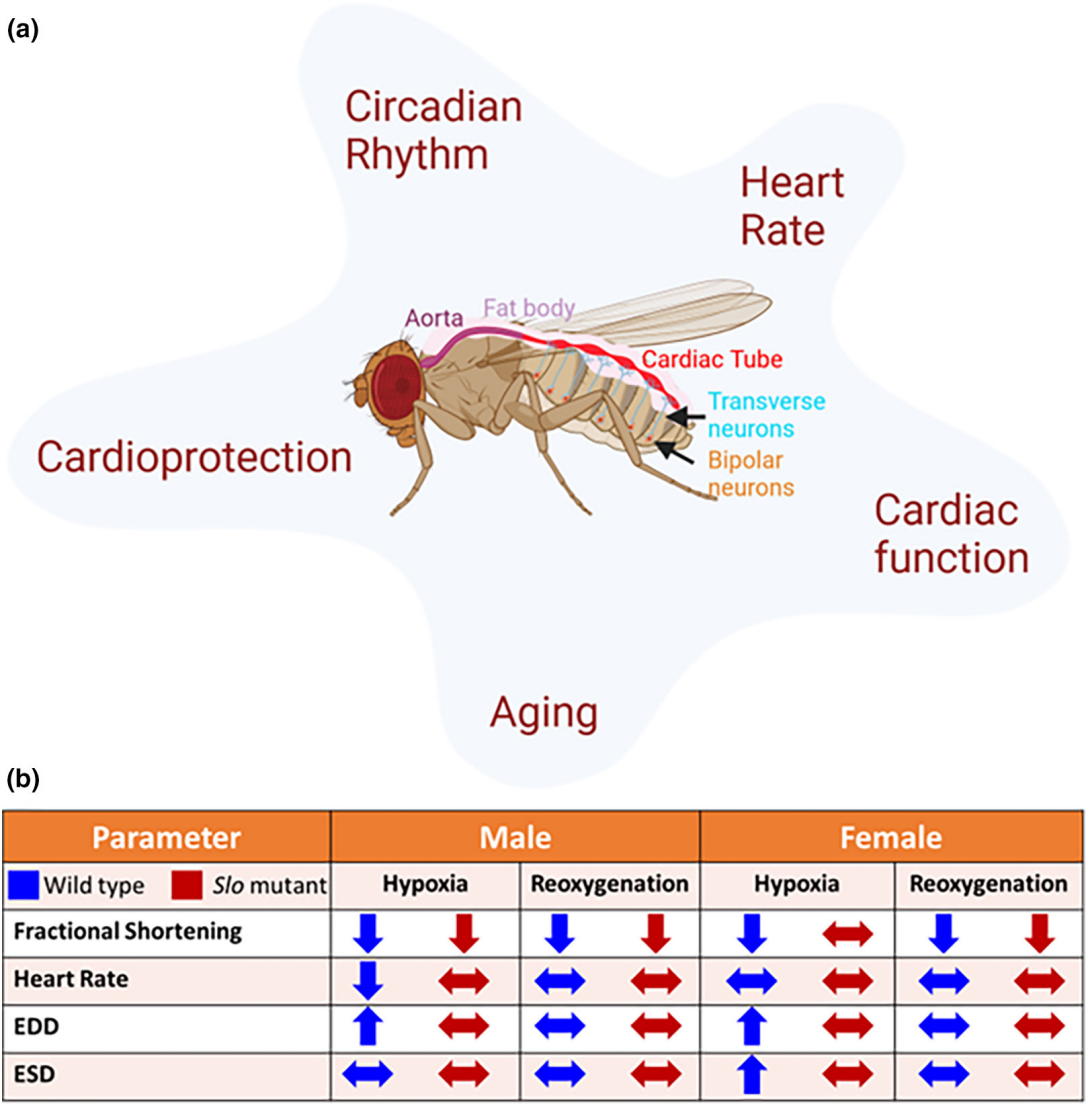
Slo in *Drosophila* cardiac physiology. (a) Schematic of role of Slo/ BK_Ca_ in *Drosophila*. Slo is known to play a direct role in cardioprotection and cardiac function. These cardiac outcomes are directly impacted by heart rate, circadian rhythm, and aging, where BK_Ca_ is known to play a significant role. The circulatory system in *Drosophila* consists of an open system with an aorta in the thorax and a cardiac tube along the dorsal aspect of the A1 abdominal segment. The cardiac tube function is controlled by transverse and bipolar neurons. (b) Schematic representation of impact of ablation of removal of *slo* on cardiac function upon IR stress. The up arrows indicate increased function, the down arrows indicate decreased function and the two‐sided arrows indicate no change as compared to the baseline function. Blue arrows are for the wild‐type and red arrows are for the *slo* mutant flies.

BK_Ca_ channels are known to play an active role in neuronal excitability (Ancaten‐Gonzalez et al., 2023; Contreras et al., 2013; Filosa et al., 2006; Meredith, 2023), circadian rhythm (Contet et al., 2016; Farajnia et al., 2015; Meredith et al., 2006; Montgomery & Meredith, 2012), cardiac function (Frankenreiter et al., 2017) including heart rate regulation (Imlach et al., 2010; Lai et al., 2014; Patel et al., 2018), and cardioprotection from ischemia–reperfusion injury (Frankenreiter et al., 2017; Goswami et al., 2018; Heinen et al., 2014; Singh et al., 2013; Szteyn & Singh, 2020) (Figure 6a). In *Drosophila*, BK_Ca_ channels are characterized for their roles in neuronal functions (Atkinson et al., 1991), aging (Gururaja Rao et al., 2019), and mitochondrial physiology (Becker et al., 1995; Elkins et al., 1986). In the heart, BK_Ca_ is highly expressed in the sinus node in humans and mice (Frankenreiter et al., 2017; Pineda et al., 2021). Inactivation of BK_Ca_ is known to affect the heart rate in rats, mice, and embryonic zebrafish (Frankenreiter et al., 2017; Patel et al., 2018; Pineda et al., 2021). A loss‐of‐function mutation in the *Kcnma1* gene is associated with tachy‐brady syndrome and atrial fibrillation (Liang et al., 2019). In *Drosophila*, earlier studies implicated BK_Ca_ in heart rate regulation in larvae (Johnson et al., 1998), but subsequent studies showed that at baseline there are no differences in the heart rate between *slowpoke* mutants and wild‐type larvae (Hensley et al., 2023). The difference could arise from the approach used for obtaining the cardiac function or the sex difference. To address this discrepancy, we used adult *Drosophila* as a model system for our studies, where cardiac function was measured on intact flies by a non‐invasive OCT approach (Lam et al., 2018; Ma et al., 2010; Wolf et al., 2006). We have observed sex‐based differences, which could again be a reason for the different results observed in previous studies. However, we have not tested how BK regulates the heart rate, given that *Drosophila* has a myogenic pacemaker and neuronal regulation of heart rate mechanisms (Figure 6). Future studies need to tease out which tissue‐specific BK is involved in the process (Andersen et al., 2015). Though *Drosophila* is a valuable model for dissecting the roles of genes that are involved in cardiac function and cardiac diseases, it is not an adequate model for circulatory diseases or the cardiac conduction system (Wolf & Rockman, 2011).

We have previously shown that *slo* mutants show early aging phenotype (Figure 6) (Gururaja Rao et al., 2019). Since we observed changes in the cardiac function in *slo* mutants in adults and other groups reported conflicting data in the larvae, we tested whether there is an age‐related cardiac function phenotype in *slo* mutants. As anticipated, *slo* mutants showed reduced cardiac function phenotypes early on and continued to show this deterioration as measured week by week. This agrees with data from mice and humans where, with age, expression of BK_Ca_ channels decreases, which results in abnormal vascular function (Albarwani et al., 2010; Carvalho‐de‐Souza et al., 2013; Kang et al., 2009; Reed et al., 2020). Given the presence of BK_Ca_ in the mitochondria of hearts, this function could be attributed to a defect in mitochondrial function in *slo* mutants. A decline in mitochondrial function and quality has been associated with normal aging as well as corroborated with age‐related cardiac dysfunction (Balaban et al., 2005; Hamilton & Terentyev, 2019; Huhn et al., 2012; Sahin et al., 2011). In agreement, our studies previously have also reported a compromised function of mitochondria in *slo* mutants (Goswami et al., 2018; Gururaja Rao et al., 2019; Singh et al., 2013). We have previously shown that combined mitochondrial oxygen consumption is significantly higher in *slo* ^1^ mutants (Gururaja Rao et al., 2019) indicating that there could be metabolic rate differences that could be contributing to the cardiac defects we observe in the *slo* mutants. Perhaps the faster‐working mitochondria in *slo* mutant hearts might be functionally ineffective.

Unlike the role of BK_Ca_ in cardiac function, the channel's role in protecting the heart from ischemia–reperfusion has been well established (Frankenreiter et al., 2017, 2018; Heinen et al., 2014; Sanghvi et al., 2022; Shi et al., 2007; Singh et al., 2013; Stowe et al., 2006; Wojtovich et al., 2013). Since we observed differences in cardiac function in males and females, we tested whether there was a sex difference in response to hypoxia and reoxygenation in flies. We observed that the *slo* mutants did not undergo as much damage as wild‐type flies upon hypoxia and reperfusion. Both male and female *slo* mutants had varied degrees of protection against hypoxia and reperfusion compared to wild‐type flies (Figure 6). Male mutant flies did undergo reduced fractional shortening, but it wasn't as significant as in wild‐type flies after hypoxia. In female flies, the damage was further reduced. On reperfusion, the recovery was similar to the wild‐type in female but not male flies. This behavior can be explained due to *slo* mutant hearts already having reduced cardiac function. The stress could trigger possible compensatory mechanisms in *slo* mutants. This kind of preconditioning might allow them to respond to hypoxic stress better than wild‐type flies, where the cardiac function is not compromised. The heart rates of *slo* mutants do not show a significant change in hypoxic stress and reoxygenation damage, again indicating that *slo* mutants are preconditioned to stress already. The sex differences could also be attributed to hormonal differences such as estrogen between males and females (Fukuma et al., 2020; Iorga et al., 2016; Kabir et al., 2015; Xu et al., 2006). *Drosophila* also express estrogen receptors, which could play a role in regulating cardiac function differentially in females (Beebe et al., 2020; Tennessen et al., 2011).

Our previous studies have shown that BK_Ca_ mutants have increased mitochondrial ROS production (Gururaja Rao et al., 2019). This could have led to compensatory pathways that handle hypoxic‐reoxygenation stress in the case of EDD and ESD. Mitochondrial ROS is known to participate in signaling events via MAPK, protein kinase‐C, and NF‐κB pathways, which can provide a beneficial effect during ischemic preconditioning. Even during postconditioning, ROS is known to participate in the signaling pathways that mediate the beneficial effects (Kleinbongard et al., 2018; Murphy & Hartley, 2018; Sun et al., 2005). Our data suggests that, though some of the functions of *slo* mutants are compromised, the flies are resistant to hypoxia‐reoxygenation injury during their short life span. While we have not measured ROS levels by staining in the hypoxia experiment, we speculate that precondition due to basal increase in ROS of *slo* mutants might explain some of their resistance to hypoxia‐reoxygenation.

One of the remarkable differences between wild‐type and *slo* mutant flies was observed in end‐diastolic and end‐systolic diameters. Immediately after hypoxia, male and female flies showed an increase in both end‐systolic and diastolic diameter, which recovered to the pre‐hypoxic levels for the wild‐type. However, in *slo* mutants, there were no observed diameter differences after hypoxia or reoxygenation. These results indicate changes in cardiac cells, which might be preventing them from undergoing morphological changes. We therefore measured *slo* mutant hearts for structural abnormalities. We measured the size of cardiac fibers using images where hearts were stained for actin fibers. As predicted, the *slo* mutant flies presented an increased thickness of cardiac fibers. This is consistent with *slo* flies having compromised cardiac function (Sessions et al., 2017; Zhu et al., 2017), a phenomenon observed in mammals extensively. It is known that ROS can induce fibrosis either through chemokines or inflammation (Richter & Keitzmann, 2016), which we speculate might be the case in *slo* mutants.

In summary, our results show that *slo* mutants compromised cardiac function and, as a result, also showed an increase in the size of cardiac fibers. However, possibly due to preconditioning due to these phenotypes, they are better equipped to face hypoxic stress. We also observe sex‐based differences in cardiac function in *slo* mutants, highlighting the fact that further studies need to be carefully done on the sexes to establish a clear role for BK_Ca_ in both males and females.

## AUTHOR CONTRIBUTIONS

S.G.R., A. L., and H.S. for cardiac function and analysis, S.G.R., S.S., J. P., and S. A., for immunocytochemistry and analysis. S.G.R., and H.S. conceptualization, supervision, project administration, and funding acquisition.

## FUNDING INFORMATION

This work is supported by National Center for advancing translational sciences (TR004178, HS) and in parts by the National Heart, Lung, and Blood Institute (HL133050 and HL157453, HS), and American Heart Association‐Transformational Project Award (965,301, HS and, 972,077, SGR). The content is solely the responsibility of the authors and does not necessarily represent the official views of the National Institutes of Health.

## CONFLICT OF INTEREST STATEMENT

The authors declare that they have no conflicts of interest with the contents of this article.

## ETHICS STATEMENT

Not applicable.

## Data Availability

All data generated and analyzed during this study are available from the corresponding author upon reasonable request.
